# Osteonecrosis of the Jaws in Patients with Hereditary Thrombophilia/Hypofibrinolysis—From Pathophysiology to Therapeutic Implications

**DOI:** 10.3390/ijms23020640

**Published:** 2022-01-07

**Authors:** Minerva Codruta Badescu, Elena Rezus, Manuela Ciocoiu, Oana Viola Badulescu, Lacramioara Ionela Butnariu, Diana Popescu, Ioana Bratoiu, Ciprian Rezus

**Affiliations:** 1 Department of Internal Medicine, “Grigore T. Popa” University of Medicine and Pharmacy, 16 University Street, 700115 Iasi, Romania; minerva.badescu@umfiasi.ro (M.C.B.); dr.popescu.diana@gmail.com (D.P.); ciprian.rezus@umfiasi.ro (C.R.); 2 III Internal Medicine Clinic, “St. Spiridon” County Emergency Clinical Hospital, 1 Independence Boulevard, 700111 Iasi, Romania; 3 Department of Rheumatology and Physiotherapy, “Grigore T. Popa” University of Medicine and Pharmacy, 16 University Street, 700115 Iasi, Romania; bratoiu.ioana3@gmail.com; 4 I Rheumatology Clinic, Clinical Rehabilitation Hospital, 14 Pantelimon Halipa Street, 700661 Iasi, Romania; 5 Department of Pathophysiology, “Grigore T. Popa” University of Medicine and Pharmacy, 16 University Street, 700115 Iasi, Romania; manuela.ciocoiu@umfiasi.ro; 6 Hematology Clinic, “St. Spiridon” County Emergency Clinical Hospital, 1 Independence Boulevard, 700111 Iasi, Romania; 7 Department of Mother and Child Medicine, “Grigore T. Popa” University of Medicine and Pharmacy, 16 University Street, 700115 Iasi, Romania; ionela.butnariu@umfiasi.ro

**Keywords:** osteonecrosis of the jaws, hereditary thrombophilia, hypofibrinolysis, anticoagulant

## Abstract

Osteonecrosis of the jaws (ONJ) usually has a clear etiology. Local infection or trauma, radiotherapy and drugs that disrupt the vascular supply or bone turnover in the jaws are its major contributors. The thrombotic occlusion of the bone’s venous outflow that occurs in individuals with hereditary thrombophilia and/or hypofibrinolysis has a less known impact on jaw health and healing capability. Our research provides the most comprehensive, up-to-date and systematized information on the prevalence and significance of hereditary thrombophilia and/or hypofibrinolysis states in ONJ. We found that hereditary prothrombotic abnormalities are common in patients with ONJ refractory to conventional medical and dental treatments. Thrombophilia traits usually coexist with hypofibrinolysis traits. We also found that frequently acquired prothrombotic abnormalities coexist with hereditary ones and enhance their negative effect on the bone. Therefore, we recommend a personalized therapeutic approach that addresses, in particular, the modifiable risk factors of ONJ. Patients will have clear benefits, as they will be relieved of persistent pain and repeated dental procedures.

## 1. Introduction

Osteonecrosis is a common pathology worldwide and has traumatic and non-traumatic causes. Within non-traumatic osteonecrosis, we distinguish the primary (idiopathic) and the secondary forms, the latter being the consequence of various local or systemic diseases, treatments or excessive alcohol consumption [1]. Attempts to explain the mechanism of primary osteonecrosis have led to the identification of coagulation and fibrinolysis abnormalities as key elements of the pathophysiological cascade. In patients with idiopathic osteonecrosis, single or multiple abnormalities of coagulation and/or fibrinolysis were identified and were considered to be responsible for the intravascular coagulation and thrombotic occlusion of bone microcirculation that lead to the ischemic bone destruction [2].

Vascular thrombosis and fat embolism as contributors to the mechanism of non-traumatic osteonecrosis were first proposed in 1934 [3], but the concept was refined in the early 1990s. It was thought that intravascular coagulation follows intraosseous fat embolism. Local vascular stasis leading to a low clearance of procoagulant factors and hypercoagulability, impaired fibrinolysis, endothelial damage induced by hypoxia and free fatty acids, and subchondral vasoconstriction were all considered contributors to the progression of fatty osteocytic necrosis to the ischemic degeneration of osteocytes and adipocytes [4]. Since the capillary and sinusoidal bed is characterized by a small vascular lumen and slow blood flow, the subchondral microcirculation is the most vulnerable area and it represents the place where the pathological process begins. Further on, progressive venous and retrograde arterial thrombosis occurs, leading to bone devascularization [4]. The mechanism behind the thrombotic occlusion of the bone’s venous outflow as the initiating mechanism of osteonecrosis was refined later on [5,6]. It was postulated that thrombotic venous obstruction leads to bone marrow edema and to a reduction in arterial perfusion. The increased intraosseous pressure determines a hypoxic-anoxic environment responsible for ischemic bone infarction and necrosis. The thrombotic mechanism was confirmed by histopathological findings represented by dilated marrow sinusoids and veins with an intravascular plug of fibrin and platelets, marrow necrosis and the focal loss of osteocytes in the adjacent bone [7].

The contribution of coagulation abnormalities in the occurrence and progression of osteonecrosis was gradually elucidated as the knowledge of the thrombotic/fibrinolytic pathways increased and technological progress allowed the laboratory quantification of coagulation factors. The data accumulated so far show that hypercoagulability and hypofibrinolysis are not uncommon in patients with osteonecrosis [2]. The majority of studies have focused on osteonecrosis of large joints due to its long-term disabling consequences. Glueck et al. showed that 74% of patients with osteonecrosis of the hip, knee or shoulder had one or more primary coagulation disorders [5]. In a group of 30 patients with osteonecrosis of the hip, coagulation disorders were identified in 87% of cases [8]. Jones et al. identified a coagulopathy in 82.2% of patients with osteonecrosis of large joints and in only 30% of controls. Moreover, two or more abnormal test results were identified in 46.7% of patients with osteonecrosis and in 2.5% of controls [2]. Based on this experience, researchers have hypothesized that hypercoagulability and hypofibrinolysis are not only contributors to avascular necrosis of the large joints—especially the femoral head—but also of the jawbones. 

The jaws are structures with many particularities. Firstly, their intramedullary environment houses marrow tissues and it is in contact with teeth and their supporting structures, thus being easily exposed to infections. Secondly, the jaws contain large sensory nerve trunks of trigeminal nerves, which explains the chronic facial or jaw pain associated with cavitational osteonecrosis, often resistant to treatment and disabling [7]. Chronic neuralgia-like facial pain is associated with intraosseous cavity formation and long-standing cancellous bone necrosis of the jaws with minimal regenerative capabilities [6]. The chronic unremitting and disabling pain is usually refractory to conventional medical and dental treatments and requires a long-term administration of analgesics, including oral narcotics. Some patients have opposite symptoms such as hypoesthesia, paresthesia or even anesthesia [9]. Symptoms are present in the territory of the inferior alveolar nerve and include numbness of the lower lip, chin, teeth and gingival mucosa. This neuropathy, also known as numb chin syndrome, was mostly related to odontogenic causes and malignancies but there are data confirming that it may also be the expression of osteonecrosis [10]. Patients were diagnosed with ONJ if they had exposed bone in the oral cavity that persisted without healing for longer than eight weeks after identification [11]. To reflect the broad spectrum of clinical manifestations as accurately as possible, the definition of this disease has been expanded and currently includes the patients with intraoral or extraoral fistula through which the bone can be probed [12]. Efforts are being made for further improvements so that patients with signs and symptoms that cannot be attributed to a local cause, such as unexplained bone pain or swelling, toothache, mobile teeth not due to periodontitis, lip dysesthesia or mandibular fracture, are investigated for ONJ even in the absence of a fistula [13,14]. Moreover, the time frame necessary for diagnosis should not be rigid but modulated on an individual basis so that the therapeutic intervention can be implemented without delay [13].

Although most available data refer to ONJ that occurs in patients with risk factors such as dental infections, tooth extraction and/or systemic treatment with antiresorptive or antiangiogenic agents [13,15], in cases with unexplained or uncontrollable jawbone pain and confirmed osteonecrosis, an underlying familial hypercoagulability and/or hypofibrinolysis state could be presumed. This pathological substrate could be considered in patients with multiple foci of osteonecrosis as well. Long-standing ischemia due to thrombosis of the veins and sinusoids is associated not only with necrosis and cavity formation in the jaw’s cancellous bone, but also with poor bone regenerative capabilities [7]. Local prothrombotic states specific to the jaw, such as odontogenic infection, endodontic failures, tooth extraction, or endothelial trauma from other alveolar bone surgery, may overlap with systemic prothrombotic conditions and favor the onset and progression of osteonecrosis [6].

Under the action of various stress factors, a reversible injury can progress to an irreversible injury, marrow cell necrosis, asymptomatic bone cell necrosis and finally to symptomatic bone cell necrosis, as the most severe manifestation form [16]. We have to consider that the ischemic threshold varies with individual characteristics, some of which are not obvious when first assessed, such as thrombophilia/hypofibrinolysis states. 

The aim of our research was to provide up-to-date and systematized information on the prevalence and importance of hereditary thrombophilia/hypofibrinolysis states on the occurrence, evolution and treatment of the osteonecrosis of the jaws (ONJ). We performed a systematic search in the Web of Science database using the keywords “osteonecrosis” and (“thrombophilia” or “hypofibrinolysis”). The automatic search retrieved 189 results that were manually screened, including their reference lists, in order to collect all available data on jaw osteonecrosis. In the final analysis, were included eight original articles focusing on hereditary thrombophilia/hypofibrinolysis in patients with ONJ. 

## 2. Etiology of the Osteonecrosis of the Jaws

ONJ is recognized as a multifactorial disease, encompassing a large variety of local and systemic causes [17]. It is more commonly found in the mandible than maxilla because the latter has better vascularity [18]. The prevalence of lesions is 65%, 28.4%, 6.5% and 0.1% in the mandible, maxilla, both mandible and maxilla, and other locations, respectively [15]. 

Radiotherapy and drugs that disrupt vascular supply or bone turnover in the jaws are major contributors to its etiology. Medication-related osteonecrosis of the jaw (MRONJ) [12] may occur in patients undergoing systemic therapy with bone-modulating, antiangiogenic or immunosuppressive drugs. Bisphosphonates and receptor activator of nuclear factor kappa-B ligand (RANKL) inhibitors are bone-modulating agents with antiresorptive effects used in both osteoporotic and metastatic cancer patients to improve bone strength and prevent fractures. In oral administration, bisphosphonates (BPs) are indicated in the treatment of osteoporosis as they decrease osteoclastic bone resorption [9]. In these patients, the prevalence of ONJ is very low, between 0 and 0.04% [15,19,20,21]. Intravenous bisphosphonate treatment is generally used in patients with metastatic cancer to stabilize the affected bone and to prevent fractures and it is given in high doses [22]. In these patients, the prevalence of ONJ reaches 0.348% [15], probably in relation to bone turnover suppression [23] and antiangiogenic properties [24] of BPs administrated in high doses. Depending on the therapy length, 1 to 10 from 100 cancer patients treated with high doses of iv BPs will develop ONJ [25,26]. RANKL inhibitors are used in patients with osteoporosis and different types of metastatic cancer (breast, lung and prostate). They block the maturation, function and survival of osteoclasts, thus decreasing osteoclast-mediated bone resorption and turnover [27]. Although developed to outperform BPs, the RANKL inhibitor denosumab caused ONJ at a similar rate, especially when other factors, such as dental extraction or chemotherapy, were concurrent [28,29].

The systemic administration of antiresorptive agents has the potential to inhibit the function of osteoclasts in the entire skeleton. Still, the remodeling response is different between craniofacial and peripheral bones and osteonecrosis occurs only in jawbones [30]. Although the site specificity of medication-induced osteonecrosis has been intensively studied, its complex mechanism is not completely elucidated yet [31]. One particular aspect is that in the jaws the healing process begins with bone resorption, while in long bones it starts with bone formation [32]; therefore, therapeutic agents that inhibit bone resorption significantly affect jaw healing. Another aspect is that the maxilla and mandible have higher rates of bone turnover compared to the long bones, and thus there is a higher uptake of BPs. So, jawbones may accumulate in high amounts of BPs that affect osteoblasts when they reach toxic levels. This leads to their decreased survival [31]. BPs act not only on osteocytes, osteoblasts and osteoclasts but also on fibroblasts, epithelial and mesenchymal stem cells. Since periodontal ligament stem cells have the potential to regenerate the alveolar bone, their suppression by BPs may contribute to osteonecrosis [33]. Moreover, increased necrotic areas and reduced vascularization in the periodontal ligament were also identified in relation to BP use [34].

The discontinuation of the antiresorptive treatment (the so-called drug holiday) was proposed as a prophylactic measure. BPs are incorporated into the mineralized bone matrix and are highly remnant in the bone. The bone clearance of BPs may extend over a period of weeks to years; therefore, it is expected that BP’s drug holiday has no or limited effects on bone turnover [35,36,37]. Still, data from animal models showed that BPs drug holiday favorably influences healing after tooth extraction. Since nitrogen-containing BPs enhances bacterial adhesion and biofilm formation, suppress angiogenesis and potentially have soft tissue toxicity, drug holiday may alleviate the bone from this burden and allow regeneration [38]. Denosumab is a fully human monoclonal antibody and is not incorporated into the bone. With a half-life of approximately 26 days, a drug holiday may be beneficial for bone turnover, especially for patients requiring extensive invasive oral surgeries [31].

Antiangiogenic agents are inhibitors of blood vessel growth and are frequently but not exclusively used in the treatment of solid cancers [39]. Their most important target is the vascular endothelial growth factor (VEGF) axis. Some agents inhibit the receptor tyrosine kinase enzymes, while others, in the form of neutralizing antibodies, block the VEGF receptors or ligands [40]. These drugs inhibit angiogenesis and lead to blood vessel loss and avascular necrosis by interfering with endothelial cell proliferation and survival. Bones can also be affected and concomitant medical comorbidities, including systemic infections, rheumatoid arthritis, diabetes, or vascular disease, increase the risk of ONJ [41]. From the large and widely used family of antiangiogenic agents, many members have already been associated with ONJ [39,42]. Among the tyrosine kinase inhibitors, axitinib [43], cabozantinib [44], dasatinib [45], imatinib [46,47], pazopanib [48], regorafenib [49], sunitinib [50,51,52] and sorafenib [53] were identified as contributors to ONJ. Several monoclonal antibodies such as adalimumab [54,55], bevacizumab [56,57], infliximab [58], rituximab [59] and romosozumab [60] were associated with jaw pain and osteonecrosis. The monoclonal antibody ipilimumab also proved to be a causative agent for ONJ through osteoclastogenesis stimulation [61]. Tocilizumab, a humanized antiinterleukin-6-receptor monoclonal antibody that inhibits interleukin-6 signaling used in the treatment of rheumatoid arthritis and in the treatment of SARS-CoV-2 infection, was associated with ONJ as well [62]. Other new drugs such as fusion proteins, mammalian target of rapamycin inhibitors, radiopharmaceuticals and selective estrogen receptor modulators were also linked to ONJ [39].

Immunosuppressants associated with ONJ are methotrexate and corticosteroids [39]. Methotrexate is not only an immunosuppressant but a disease-modifying antirheumatic drug as well. Recently, two cases of ONJ associated with its use were reported [63]. Corticosteroids are widely used due to their immunosuppressant and anti-inflammatory potential, but long-term treatment with high doses is related to both osteoporosis and osteonecrosis. Corticosteroids influence bone health through multiple mechanisms such as decreasing the activity of bone marrow stem cell pool, bone matrix degeneration, fat emboli, hypercoagulability, vascular endothelial dysfunction, decreased angiogenesis, elevated vasoconstriction and apoptosis of osteoblasts and osteocytes [64]. Long-term high-dose steroids can act as sole or major cause of ONJ [65,66] or as aggravating factor if prescribed in combination with antineoplastic drugs. As corticosteroids are often part of the oncological therapeutic regimens, there is a debate as to which of the two drugs has a primary role in bone necrosis and which is a secondary one, exacerbating this phenomenon.

Osteoradionecrosis (ORN) follows radiation therapy to the head and neck of cancer patients. Its reported incidence varies between 2 and 22% and usually occurs two to four years after the completion of radiation treatments [67]. It can be spontaneously or induced by dental extractions and dental implants. The radiation-induced fibrosis theory is the most accepted mechanism for ORN. The pathophysiological cascade begins with the formation of free radicals, endothelial dysfunction, inflammation, microvascular thrombosis and ends with fibrosis, remodeling, and finally bone necrosis [68,69]. Due to advances in radiotherapy delivery techniques, the number of ORN cases is currently low [70].

Systemic diseases such as rheumatoid arthritis and diabetes mellitus are important substrates for osteonecrosis. In patients with rheumatoid arthritis, ONJ occurrence is usually correlated with BP use [31]. Diabetes mellitus is a metabolic condition known for the delay in wound healing. The bone becomes vulnerable through multiple mechanisms such as endothelial cell dysfunction, microvascular ischemia, increased apoptosis of osteoblasts and osteocytes and reduced bone remodeling [31,71].

Oral infections (e.g., untreated caries, pulp infections, periodontal disease, and osteomyelitis) and trauma or injury to the jawbones, including dentoalveolar surgery, were also associated with ONJ [70,72]. They can act independently or potentiate the harmful effect of systemic factors. The cumulative action of antiresorptive therapy with tooth infection and extraction has the strongest negative effect on the bone [13]. The high cumulative dosage of nitrogen-containing BPs and the presence of tooth infection rather than extraction were most correlated with ONJ occurrence [73,74]. Periodontal and periapical infections have the potential to alter the number and function of osteoclasts and lead to ONJ, whether or not they are associated with tooth extraction [31].

Other etiologies of ONJ are rare. Disseminated intravascular coagulation (DIC) generating occlusive thrombi in intraosseous vessels of the maxilla was considered the mechanism that led to bone necrosis in an 83-year-old patient with a recent history of sepsis and DIC induced by acute myeloid leukemia [75]. Sickle cell disease, an inherited condition characterized by increased blood viscosity and vaso-occlusive crises due to abnormal erythrocytes shape, was considered responsible for osteonecrosis in the mandible in a 39-year-old patient [76]. The causality between alcoholism, malnourishment and ONJ was also highlighted [77]. In one patient, Gaucher’s disease was considered the cause of ONJ [78]. Gaucher’s disease is a glycolipid storage illness caused by the genetic deficiency of the lysosomal enzyme beta-glucocerebrosidase, resulting in the accumulation of glucosylceramide in the macrophages. This abnormal cell may infiltrate the bone marrow and lead to bone ischemia and necrosis.

On rare occasions, none of the causes mentioned above can be identified. Based on the observation that arterial and venous thrombotic events are more frequent in individuals with coagulation and/or fibrinolysis disorders [79,80], and considering the hypothesis of thrombotic occlusion of intramedullary veins as the initiating event that leads to impaired intraosseous circulation and osteonecrosis [5], it seems reasonable to search a hypercoagulability and/or hypofibrinolysis state in patients with osteonecrosis whose etiology remained unknown.

## 3. Hereditary Thrombophilia in Patients with ONJ

Thrombophilia includes inherited or acquired disorders that increase a person’s risk of developing arterial and venous thrombosis. The most common and important hereditary thrombophilia are the Factor V Leiden, G20210A polymorphism in the prothrombin gene, deficiencies in protein C, protein S and antithrombin III, and the methylenetetrahydrofolate reductase C677T gene polymorphism [81,82,83]. Studies aiming at the identification of a prothrombotic substrate in patients with venous thromboembolism (VTE) showed a strong association between coagulation abnormalities and VTE occurrence. In 35–61% of VTE patients, a hereditary thrombophilic defect can be detected [84,85,86]. Moreover, the younger the patients are at their first VTE episode, the more likely they are to have a coagulation abnormality. A prior history of VTE is also an indicator for the presence of a thrombophilic substrate [85,87]. While inherited thrombophilia is a well-established predisposing factor for VTE, its role in arterial thrombosis has remained a long-lasting uncertainty. However, a recent meta-analysis found a strong association between inherited thrombophilia (Factor V Leiden, G20210A polymorphism in the prothrombin gene, protein C and protein S deficiencies) and the increased risk of arterial ischemic stroke in adults [88]. Recently, high levels of factors VIII (FVIII) [89,90,91] and von Willebrand (vWF) [92,93] were associated with an increased risk of both arterial and venous thrombotic events.

### 3.1. Factor V Leiden

Factor V (FV) has a major role in the coagulation cascade, serving as a cofactor for factor X (FX). Activated FV (FVa) binds to activated FX (FXa) and forms the prothrombinase complex, which converts large amounts of prothrombin to thrombin, leading to fibrin generation. FXa can activate prothrombin even in the absence of its cofactor, but when assembled into the prothrombinase complex along with FVa, it increases the prothrombin conversion rate by ~10,000 times [94]. FV Leiden is an abnormal protein determined by a single-nucleotide polymorphism (1691G>A) in the FV gene. Its function is not altered, but the rate at which it is inactivated by activated protein C is significantly reduced, leading to a prothrombotic state [95].

FV Leiden is the most frequent cause of inherited thrombophilia. Its prevalence in the general population is 5% [96], the highest being in Caucasians and the lowest in Asians [97,98,99]. A lot of evidence of its contribution to the idiopathic osteonecrosis of the hip and knee has been found. Of 161 North American patients with idiopathic osteonecrosis of the femoral head (ONFH), 9.3% had FV Leiden [100]. In another study, heterozygosity for FV Leiden was identified in 8 out of 35 patients with idiopathic ONFH [101]. A European study enrolling 72 patients with ONFH and 300 healthy volunteers showed that patients with ONFH were 4.5 times more likely to have FV Leiden than the controls. Furthermore, patients with idiopathic ONFH were 5.7 times more likely to have FV Leiden than patients with secondary ONFH [102]. Similar, from 38 patients with osteonecrosis of the knee 11 (29%) had FV Leiden [103]. 

Evidence of FV Leiden presence in patients with osteonecrosis of the jaw exists as well (Table 1). Pandit et al. published the case of a 55-year-old Caucasian male with osteonecrosis of both mandible and maxilla, and facial pain after 6 months on testosterone–anastrozole treatment [104]. Since the patient had a family history of deep vein thrombosis (including one case related to estrogen-progestin oral contraceptive use) and a personal history of four myocardial infarctions in the previous 5 years treated with multiple stents, a thrombophilic substrate was presumed. The patient was found to have multiple coagulation and fibrinolysis abnormalities: FV Leiden heterozygosity, 4G/4G PAI-1 homozygosity, MTHFR C677T homozygosity, and high levels of beta-2 glycoprotein and anticardiolipin antibody. Moreover, the determinations made within the patient’s family showed the presence of FV Leiden heterozygosity in the patient’s two sons and his daughter.

Jarman et al. published the case of a 32-year-old man that had 10 years of unexplained tooth loss, progressing to primary ONJ with cavitation 8 months after starting testosterone [105]. Its coagulation studies revealed mixt defects: FV Leiden heterozygosity, 4G/4G PAI-1 homozygosity and lupus anticoagulant.

**Table 1:** Studies on hereditary thrombophilia/hypofibrinolysis in patients with ONJ.

Author, Year	No. of Patients	Type of Defects	Hereditary Thrombophilia/Hypofibrinolysis Traits–No. of Patients			Acquired Thrombophilia	Comments
			Thrombophilia (Only)	Hypofibrinolysis (Only)	Thrombophilia + Hypofibrinolysis (Mixed)		
Gruppo et al., 1996 [106]	55	Single Hereditary Defect (13 patients)	APCR–2 ↓Prot C–1	↑Lp(a)–5 ↓tPA–4 ↑PAI-1–1			12 patients were normal 8 patients had only ACLA
		Multiple Hereditary Defects (12 patients)		↑Lp(a) + ↓tPA–2 ↑Lp(a) + ↑PAI-1–2 ↑Lp(a) + ↓tPA + ↑PAI-1–1	APCR + ↓tPA–1 APCR + ↑PAI-1–2 APCR + ↑Lp(a)–1 ↓Prot C + ↑Lp(a)–1 ↓Prot S + ↑Lp(a) + ↓tPA–1 ↓Prot S + ↑Lp(a) + ↑PAI-1–1		
		Combined Hereditary + Acquired Defects (10 patients)	APCR–2	↓tPA–1 ↑Lp(a) + ↑PAI-1–1 ↑Lp(a) + ↓tPA + ↑PAI-1–1 ↓tPA + ↑PAI-1–1 ↑Lp(a)–3	ACPR + ↑Lp(a)–1	ACLA	
Glueck et al., 1996 [7]	49	Single Hereditary Defect (25 patients)	APCR–7 ↓Prot C–3	↑Lp(a)–8 ↓tPA–7			14 patients were normal
		Multiple Hereditary Defects (10 patients)	APCR + ↓Prot C–2	↑Lp(a) + ↓tPA–1	APCR + ↓tPA–2 APCR + ↑Lp(a)–2 APCR + ↓tPA + ↑Lp(a)–1 ↓Prot C + ↑Lp(a)–1 ↓Prot C + ↓Prot S + ↑Lp(a)–1		
Glueck et al., 1997 [107]	89	Single Hereditary Defect (21 patients)	heterozygosity for the FV Leiden 16/76 women 5/13 men	Not assessed			Exogenous estrogen therapy increases the risk of ONJ
Glueck et al., 1998 [108]	1	Single Hereditary Defect	heterozygosity for the FV Leiden				Exogenous estrogen therapy increases the risk of ONJ
Vairaktaris et al., 2009 [109]	1	Single Hereditary Defects	prothrombin G20210A				BPs treatment for 5 years for osteolytic lesions related to cancer ONJ occurred 3 years after cessation of BPs therapy, after dental extraction
Pandit et al., 2014 [104]	1	Combined Hereditary + Acquired Defects			FV Leiden heterozygosity MTHFR C677T homozygosity 4G4G PAI-1 homozygosity	beta 2 glycoprotein IgM, ACLA IgM	Therapy with anastrozole and testosterone
Jarman et al., 2017 [105]	1	Combined Hereditary + Acquired Defects			heterozygosity for the FVL mutation homozygosity for the PAI-1 4G/4G mutation	lupus anticoagulant	Exogenous testosterone therapy increase the risk of ONJ

APCR = activated protein C resistance; FV = coagulation factor V; Prot C = protein C; Prot S = protein S; Lp(a) = lipoprotein(a); tPA = tissue plasminogen activator; PAI-1 = plasminogen activator inhibitor 1; MTHFR = methylenetetrahydrofolate reductase; ACLA = anticardiolipin antibody; ONJ = osteonecrosis of the jaw; BPs = bisphosphonates.

Glueck et al. published the case of a 32-year-old woman with alveolar osteitis after a routine mandibular first molar extraction with unfavorable evolution toward osteonecrosis and severe alveolar neuralgia requiring iv morphine [108]. Her genetic testing revealed heterozygosity for FV Leiden. As she had been under exogenous estrogen for 1 year, it was speculated that this drug exacerbated the thrombophilia produced by the FV Leiden mutation. 

The largest study on FV Leiden prevalence in patients with ONJ included 89 patients [107]. They had ONJ and chronic jaw/facial pain resistant to conventional medical and dental treatments. The study found that 24% of patients were heterozygous for the FV Leiden mutation [107]. Additionally, FV Leiden was more prevalent in patients with ONJ (21% of women and 38% of men) than in healthy controls (3% of women and 3.7% of men). The enhancing effect of exogenous estrogens on the hereditary hypercoagulability state was also pointed out. The exogenous estrogen administration before ONJ development was identified in 81% of women with FV Leiden and in only 38% of women with a normal FV genotype, which confirmed that elevated estrogen levels due to exogenous intake (oral contraceptives or postmenopausal supplementation) increasing the thrombotic risk in women with FV Leiden [107]. 

All the data available so far support the association between FV Leiden mutation and osteonecrosis, including ONJ. When another prothrombotic factor, such as exogenous estrogen or testosterone, overlaps the risk of ONJ increases even more.

### 3.2. Protein C and Protein S Deficiencies and Activated Protein C Resistance

Proteins C and S are essential in the control of FVa and FVIIIa activity. The binding of thrombin to thrombomodulin on the endothelial cell surfaces leads to protein C activation. Functioning as an anticoagulant enzyme, activated protein C (APC) inactivates FVa and FVIIIa on negatively charged phospholipid membranes, with additional enhancement from its cofactor, protein S. It is well known that FVa and FVIIIa are powerful coagulation activators. Entering the prothrombinase complex FVa enhances the factor Xa capacity to convert prothrombin to thrombin by five orders of magnitude [94]. Moreover, FVIIIa enhances FXa generation by 200,000-fold by entering the tenase complex [110]. Therefore, the inhibitory effect of APC is a very important regulatory mechanism of the thrombin generation. 

If any of proteins C and S is deficient, the FVa and FVIIIa are inadequately suppressed, leading to a hypercoagulability state. Deficiencies in proteins C and S each have an incidence of about 0.1% in the general population [111,112]. A hypercoagulability state may appear in the presence of FV Leiden and the antiphospholipid antibody syndrome as well. Due to its abnormal structure, FV Leiden inactivation by APC is impaired, favoring thrombosis. Activated protein C resistance (APCR) may be hereditary—in the presence of FV Leiden—or acquired—the most common cause being the antiphospholipid antibody syndrome. The presence of autoantibodies directed against phospholipids surfaces impairs the thrombomodulin–protein C–protein S anticoagulant system [113], thus creating an environment prone to thrombosis. Before a cDNA assay for FV mutation become available, the data were reported in general as APCR [7].

Deficiencies in proteins C and/or S have already been linked to ONFH. In patients with idiopathic ONFH, protein C and protein S deficiencies were present in 29.4% and 5.9% of cases, respectively, while in patients with secondary ONFH, these deficiencies were present in 21.6% and 11.8% of cases, respectively [114]. One study found that low protein C and protein S levels were more frequently encountered in patients with idiopathic ONFH than with secondary ONFH. Patients with idiopathic ONFH were 2.14 times more likely to have protein C deficiency and five times more likely to have a protein S deficiency than patients with secondary ONFH [115]. In 535 patients with ONFH, 15.5% of patients had an FV Leiden mutation and/or APCR [116]. Korompilias et al. found that 33% of patients with ONFH had APCR [117].

In a study of Glueck et al., 42 women and 7 men with idiopathic ONJ and severe chronic jaw or facial pain syndromes that were uncontrollable with standard medical and dental treatments were included [7]. Long-term pain relief with narcotics including oral morphine sulphate and methadone was used by 80% of patients. In 41% of cases, the chronic facial or jaw pain was self-reported as totally disabling. Defects in coagulation, fibrinolysis or both have been identified in 71% of cases (35 patients). Protein C deficiency was identified in seven patients. In three cases, it was an isolated anomaly, while in the remaining four cases, it was associated with APCR or hypofibrinolysis [7]. APCR was identified in 14 patients. It was a solitary anomaly in seven cases and associated with protein C deficiency or defects in fibrinolysis in the remaining seven patients. From the 10 patients with combined defects, APCR was present in seven cases. It should be noted that in the 49 patients of the study group, 29% had APCR and 14% had a low level of protein C, while none of the healthy controls had an APCR or protein C deficiency [7]. This study thus proved the association between these two heritable thrombophilic conditions and ONJ (Table 1). Furthermore, the study showed that when supplemental estrogen overlaps with APCR, the prevalence of ONJ increases. In the study group, exogenous estrogen intake was identified in 58% of women with APCR and in only 18% of women without thrombophilic/hypofibrinolytic traits. It was a confirmation of the hypothesis that estrogenic treatment amplifies the APCR-mediated tendency for thrombosis and facilitates the occurrence of osteonecrosis [7]. 

Additional data are provided by a study on 55 patients with the avascular necrosis of the jaw and chronic facial pain. In this group, 16% of patients had APCR, 4% had a protein C deficiency and 4% had a protein S deficiency [106]. In a larger group of 124 patients with ONJ and severe facial pain, 73% had abnormal coagulation–fibrinolysis parameters. APCR, protein C and protein S deficiencies were found in 17%, 6% and 3% of cases, respectively [7,106]. From the 10 patients with thrombophilia enrolled in a study evaluating the effectiveness of anticoagulant therapy on ONJ-associated pain, two had a protein C deficiency and five had APCR and/or FV Leiden [118]. We may conclude that the available evidence supports the contribution of protein C and protein S deficiencies and of APCR to the pathophysiology of ONJ.

### 3.3. G20210A Polymorphism in the Prothrombin Gene

Prothrombin G20210A is an abnormal protein determined by a single-nucleotide polymorphism (20210G > A) in the gene encoding prothrombin, resulting in an elevated plasma prothrombin level and increased thrombin generation. After FV Leiden, it is the second most common cause of inherited thrombophilia. However, the relation between the G20210A polymorphism in the prothrombin gene and osteonecrosis is not fully elucidated. However, prothrombin G20210A alone was not a significant risk factor for ONFH in North American [100] and most of the European studies [101,119], while in the Asian population this polymorphism was absent [120]. Just one European study found a high prevalence of prothrombin G20210A (8.7%) in patients with idiopathic ONFH [102]. Moreover, prothrombin G20210A was identified in only 3 of 38 patients with osteonecrosis of the knee (ONK) [103].

A single case reported the presence of G20210A polymorphism in the prothrombin gene in relation to ONJ (Table 1). It was a 72-year-old Caucasian woman, with a non-healing extraction socket after multiple attempts of medical and surgical treatments [109]. This patient had previously received (for 5 years) BP treatment for osteolytic lesions in the ribs related to cancer and ONJ occurred three years after cessation of BP therapy. Genetic testing was positive for the G20210A polymorphism in the prothrombin gene. It was concluded that ONJ was caused by overlapping of the PB on the thrombophilic substrate.

### 3.4. MTHFR C677T Gene Polymorphism

Methylenetetrahydrofolate reductase (MTHFR) is an essential enzyme in folate and homocysteine metabolism. The 677C→T polymorphism in the MTHFR gene is the most common genetic cause of hyperhomocysteinemia and recently emerged as a risk factor for cardiovascular disease and VTE [121,122]. A change in the MTHFR gene leads to disturbances in homocysteine metabolism and its accumulation into the blood. In conditions of elevated plasma levels, homocysteine oxidizes itself and produces reactive oxygen species that impair endothelial cells’ structure and function. The imbalance created leads to vasoconstriction and thrombosis [123].

The MTHFR C677T gene polymorphism’s role in osteonecrosis is still under debate. While some studies positively correlated the MTHFR C677T gene polymorphism with ONFH [102,119,120,124], others did not confirm this association [125,126]. Similarly, the relationship between the polymorphism of the MTHFR C677T gene and ONK has little and contradictory evidence [127,128]. 

The MTHFR C677T gene polymorphism was found in only one patient with ONJ [104]. It was the case of a 55-year-old male with multiple thrombophilia/hypofibrinolysis traits, who developed ONJ after testosterone–anastrozole therapy (Table 1). Thus, the role of the MTHFR C677T gene polymorphism in the pathophysiology of ONJ requires further studies. 

## 4. Hypofibrinolysis Associated with ONJ

Hereditary hypofibrinolysis is found in less than 1% of the general population, in 5–15% of deep vein thrombosis patients and in 18–22% of patients with osteonecrosis of the hips, knees and jaws [129]. The major regulators of fibrinolysis are tissue plasminogen activator (tPA) and plasminogen activator inhibitor 1 (PAI-1). While tPA is the major stimulator of fibrinolysis, PAI-1 is its main inhibitor. As PAI-1 activity accounts for about 60% of the activity of inhibitors, its overexpression promotes thrombotic events [130]. Elevated PAI-1 levels are a risk factor for VTE (especially in Asians [100]), myocardial infarction [131] and stroke [132].

Lipoprotein (a) (Lp(a)) is a contributor to hypofibrinolysis as well. It has proatherogenic, prothrombotic, and antifibrinolytic properties [133,134]. Elevated Lp(a) levels are a genetic risk factor for atherosclerosis and coronary heart disease [135]. Furthermore, elevated levels of Lp(a) are associated with an increased risk of stroke [136] and myocardial infarction [137,138]. However, the association between Lp(a) and VTE is not yet well clarified. While a meta-analysis and several other studies show a significant association between high levels of Lp(a) and increased risk of VTE in adults [139,140,141,142], other studies did not confirm this association [143,144,145].

### 4.1. Tissue Plasminogen Activator and Plasminogen Activator Inhibitor 1

TPA is a serine protease released from endothelial cells that catalyzes the conversion of plasminogen to plasmin, which is the primary enzyme involved in thrombus dissolution. PAI-1 is synthesized and released by endothelial cells as well. It is a serine protease inhibitor that exerts its function by binding to tPA and reducing tPA’s ability to convert plasminogen to plasmin. Elevated plasma levels of PAI-1 are responsible for the decreased fibrinolytic activity and correlate with the development of arterial and venous thrombosis [130,146,147]. A single guanosine nucleotide insertion/deletion variation at bp2675 of the PAI-1 promoter is responsible for the 4G/5G polymorphism. Of its three genotypes (4G4G, 4G5G, and 5G5G) the 4G4G genotype is the most associated with high plasma PAI-1 activity and, by consequence, with thrombotic events [130,147].

To date, three large meta-analyses confirmed the relation between the 4G/5G polymorphism of the PAI-1 gene and the susceptibility to ONFH. The meta-analysis of Liang et al. of five studies confirmed the presence of an increased risk of ONFH in the 4G4G genotype carriers, especially among Caucasians [148]. The meta-analysis of Zeng et al. of five randomized controlled trials revealed that 4G allele carriers have a 1.76 times higher risk of ONFH than 5G allele carriers [149]. Sobhan et al. conducted a meta-analysis of six studies and confirmed the association between the PAI-1 4G/5G polymorphism and the risk of ONFH in both Caucasians and Asians [150]. Although scarce, data regarding the association between the 4G/5G polymorphism of the PAI-1 gene and ONK are available as well. 4G4G homozygosity of the PAI-1 gene was reported in two patients with stage II knee osteonecrosis enrolled in a small study evaluating the effectiveness of the anticoagulant treatment on the progression of ONK [127]. 

One early study reported hypofibrinolysis with low stimulated tPA activity in 11 of the 49 patients with idiopathic ONJ and severe chronic jaw or facial pain syndromes, uncontrollable with standard medical and dental treatments [7]. Four of them had concurrent APCR and/or high Lp(a) levels (Table 1). 

In 124 patients with ONJ, low stimulated tPA activity was found in 22% cases and high PAI-1 activity in 19% cases [107]. Similar, in 55 patients with ONJ and severe facial pain, significantly more hypofibrinolysis traits were found than in controls [106]. Low stimulated tPA was found in 22% of cases and 7% of controls, and high PAI-1 activity in 18% of cases and 8% in controls. In a pilot study of the treatment of thrombophilia and hypofibrinolysis, including 20 patients with ONJ and hypofibrinolysis, 10 patients had high levels of PAI-1, low levels of tPA, or both [118].

Data are provided from isolated case reports as well. A 55-year-old patient with a history of multiple coronary events and ONJ following testosterone–anastrozole treatment was found with mixed defects, including 4G4G homozygosity of the PAI-1 gene [104]. PAI 4G/4G homozygosity in association with two other prothrombotic defects was also identified in a 32-year-old male with ONJ taking testosterone [105].

### 4.2. Lipoprotein (a)

Lp(a) is a low-density lipoprotein consisting of a lipid core and two apolipoproteins: apo(a) and apo(b). The apo(a) size polymorphism is the major determinant of Lp(a) levels. Most individuals have two different-sized apo(a) isoforms, each inherited from one parent, and those with small apo(a) isoforms have high plasmatic Lp(a) concentrations [138]. Lipoprotein (a) has a strong structural resemblance with plasminogen; therefore, apo(a) can bind to the sites available for plasminogen at the surface of fibrin. Because fibrinolysis is initiated on the fibrin surface by activation of cloth-bound plasminogen by tPA, fibrin-bound Lp(a) impairs fibrinolysis [151].

High Lp(a) levels were found in patients with both ONFH and ONK. Glueck et al. highlighted that hypofibrinolytic levels of Lp(a) may trigger thrombotic venous occlusion in bone microcirculation leading to osteonecrosis [5]. They identified high levels of Lp(a) in 3 of 12 patients (25%) with idiopathic ONFH and in 4 of 18 patients (22%) with secondary ONFH [8]. In another study, of 18 patients with idiopathic ONFH or ONK, 12 had high Lp(a) levels, of which three had low tPA, and, of 13 patients with secondary ONFH or ONK, four had high Lp(a) levels [5]. Korompilias et al. identified increased levels of Lp(a) in 27.3% of 216 patients with ONFH [117]. A published report of 240 patients with primary ON of either the hip or knee versus 110 healthy normal controls offered the most comprehensive analysis of the prevalence of elevated levels of Lp(a), which was higher in cases (30%) than controls (20%) [100,105,152]. However, not all available data support the association between high Lp(a) levels and osteonecrosis [2,153].

In 35 patients with ONJ and thrombophilia/hypofibrinolysis states, Glueck et al. [7] identified 14 patients with high Lp(a) levels (Table 1). In eight cases, it was the only prothrombotic trait, while in the remaining six cases, high Lp(a) levels were associated with APCR or protein C deficiency or low tPA levels [7]. In a study enrolling 55 patients with ONJ and severe facial pain at least one hypofibrinolysis trait was found in 40 patients [106]. High Lp(a) levels were identified in 36% of patients in the whole group and in 50% of patients with hypofibrinolysis. In another study, on 20 patients with hypofibrinolysis, 13 had high Lp(a) levels [118].

The available data show that high Lp(a) levels are frequently encountered in patients with osteonecrosis (hip, knee and jaw). Rarely alone, but most often in association with other thrombophilia/hypofibrinolysis traits, the high Lp(a) levels are certainly contributors to this disease. 

## 5. Treatment of Hereditary Thrombophilia/Hypofibrinolysis

Patients with hereditary thrombophilia and/or hypofibrinolysis have an increased risk for arterial and venous thrombosis. Since the thrombotic occlusion of intraosseous veins and microcirculation is considered the first event on the path leading to bone osteonecrosis, the treatment should aim to correct the state of hypercoagulability and/or hypofibrinolysis.

Studies on primary ONFH in adults showed that the treatment of thrombophilia/hypofibrinolysis has the capacity to improve symptoms and functional status and slow down the progression of osteonecrosis, even to reverse it. The mandatory condition is that the therapy is started before irreversible bone damage occurs. Several studies that included patients with ONFH in Ficat stages I-II [105,116,154,155,156,157,158,159] demonstrated that anticoagulant treatment initiated before the segmental collapse of the femoral head has beneficial effects on preserving the hip joint, alleviating the pain and improving the joint functional capabilities. 

Favorable results were reported in patients with ONK and hereditary thrombophilia/hypofibrinolysis as well. A small study enrolled six patients with stage II ONK, all with thrombophilia, and six with concurrent hypofibrinolysis [127]. Anticoagulant treatment (enoxaparin) given for at least 3 months prevented bone collapse and progression to severe osteoarthritis. Moreover, most patients registered a resolution of pain and restoration of full function. Haydock et al. published the case of a 40-year-old Caucasian woman with hypofibrinolysis (4G4G genotype of the PAI-1 gene) and osteonecrosis in the distal femur, in which the anticoagulant treatment led to the cessation of bone pain and prevented osteonecrosis progression [160]. 

Long-term anticoagulation proved to be effective in thrombophilic patients with early (pre-collapse) primary ON of the knee [127,159] and hip [105,116,154,155,156,157,158,159]. Starting from these data, it was hypothesized that by correcting the coagulation disorders in patients with ONJ, it would be possible to alleviate the facial pain and allow bone healing.

The reference study on the effect of targeted therapy on hypercoagulability and/or hypofibrinolysis in patients with ONJ was performed by Glueck et al. [118]. The study enrolled 26 patients, all suffering from disabling facial pain refractory to conventional medical and dental treatments (endodontic therapy, dental extraction, and surgical curettage of the diseased bone). Of the 26 patients, 6 had thrombophilia alone, 16 had hypofibrinolysis alone and 4 had both thrombophilia and hypofibrinolysis. The thrombophilic patients received anticoagulant treatment with a vitamin K antagonist (VKA) for 4 months, with INR targeted to 2.5–3. The patients with hypofibrinolysis were treated with 6 mg of stanozolol per day (an anabolic-androgenic steroid). Patients with thrombophilia and hypofibrinolysis received a particularized treatment protocol represented by VKA alone followed by stanozolol alone, at least 4 months apart. The study assessed the extent to which therapy specific to states of hypercoagulability/hypofibrinolysis alleviates facial pain. Pain relief was achieved in 60% of patients taking VKA and in 60% of patients taking stanozolol, usually after 8 to 12 weeks of treatment. It was noted that if pain relief was not reached in the first 12 weeks of treatment, the continuation of treatment until 28 weeks provided no incremental benefit. Only one patient on VKA had stopped therapy after 28 weeks because of nosebleeds, while stanozolol treatment was associated with side effects in 70% of patients, which were reversed within six weeks of stopping stanozolol.

## 6. Discussion

Osteonecrosis is a common disease, with a broad etiological spectrum, diverse localization in the skeleton and severe long-term consequences (impaired mobility and/or chronic pain). In most situations, the etiology can be easily established by identifying the presence of those factors that are recognized as causing bone damage. Along with local factors [70,72], the main contributors to ONJ are drugs and radiation therapy to the head and neck [12,39,67].

Despite the sustained effort to find the cause of bone destruction, in some situations, the etiology could not be determined. The hypothesis of thrombotic occlusion of bone microcirculation emerged as a plausible pathophysiological mechanism of osteonecrosis. As systemic arterial and venous thrombotic events can occur in the context of coagulation and fibrinolysis abnormalities, their contribution to the initiation and progression of osteonecrosis was extensively investigated. Conditions capable of triggering intravascular coagulation include familial thrombophilia, hyperlipemia, hypersensitivity reactions (allograft organ rejection, immune complexes, and antiphospholipid antibodies), bacterial endotoxic reactions and various viral infections, proteolytic enzymes (pancreatitis), tissue factor release (inflammatory bowel disease, malignancies, neurotrauma, and pregnancy), and other prothrombotic and hypofibrinolytic conditions [161].

Thrombophilia and hypofibrinolysis are common conditions in patients with osteonecrosis, one or both being found in up to 76% of cases [118]. Moreover, the hereditary thrombophilia/hypofibrinolysis state is nowadays recognized as an important contributor to the osteonecrosis of the hip and knee. While much evidence has been provided supporting the involvement of FV Leiden [100,101,102,103], deficiencies in protein C and/or S [114,115], APCR [115,116] and high FVIII levels [162,163,164,165] in the pathogenesis of ONFH and ONK, other hereditary anomalies of coagulation possibly have no influence (e.g., the 20210A polymorphism in the prothrombin gene [100,101,119,120]) or show contradictory results (e.g., the MTHFR C677T gene polymorphism [102,119,120,124,125,126]) in relation to the osteonecrosis of large joints. The hereditary hypofibrinolysis traits strongly associated with the osteonecrosis of the hip and knee were 4G4G homozygosity of the PAI-1 gene [127,148,149,150] and, in some studies, high Lp(a) levels [100,105,152].

The presence of a similar association between osteonecrosis and hereditary thrombophilia/hypofibrinolysis was investigated in patients with ONJ, refractory to medical and surgical treatment, associated or not with chronic facial pain. Hereditary thrombophilia was found in 50–70% of patients with osteonecrosis of the hips, knees and jaws [129]. In one study, the thrombophilic/hypofibrinolytic defects—isolated or in association—were identified in 78% of patients with ONJ [106].

Most evidence supports the contributing role of FV Leiden in ONJ [104,105,107,108]. While in patients with primary osteonecrosis of the femur and knee it may be the only identifiable thrombophilic trait [100,102], FV Leiden is rarely the sole determinant of ONJ. Patients either have multiple traits that lead to a prothrombotic state [104,105] or undergo treatments with the potential to tip the hemostatic balance toward thrombosis [107,108]. The potentiating role of hormone therapy seems to be the most important, as ONJ in FV Leiden carriers and, more generally, in patients with APCR was associated with estrogen therapy [7,107,108]. 

Exogenous estrogen has the potential to increase the risk of both arterial [166,167] and venous thrombosis [168] by interfering with coagulation and fibrinolysis at multiple levels. Estrogen enhances coagulation by elevating the plasma levels of factors II, VII, VIII, X, fibrinogen and vWF, and by decreasing the plasma levels of natural inhibitors of hemostasis, such as tissue factor pathway inhibitor, antithrombin III and protein S. Studies have shown that patients taking estrogen have significantly higher APCR levels than estrogen non-takers [169]. It also affects fibrinolysis by decreasing PAI-1 levels and increasing tPA levels. The thrombotic risk is high in the first year of treatment [170] and although it decreases after treatment discontinuation, it may persist for several years [170]. The role of estrogen in the pathogenesis of ONJ as a potentiating factor of a hereditary state of hypercoagulability is very plausible, especially since its use has been associated with thrombosis in unusual sites—cerebral venous sinus thrombosis [171,172], mesenteric venous thrombosis [173] and retinal vein occlusion [174]. Exogenous estrogen-induced thrombophilia superimposed on familial thrombophilia increased the likelihood of thrombotic events, not only in relation to osteonecrosis of the jaw [7,106,107], but also of other bones [175,176].

There is evidence that in the presence of combined coagulation and/or fibrinolysis defects, exogenous testosterone therapy has the potential to increase the risk of ONJ. Only two case reports confirm this hypothesis [104,105]. These results are in line with the data from studies focusing on large-joint osteonecrosis in patients with hereditary thrombophilia/hypofibrinolysis. Hip–knee osteonecrosis that developed after testosterone therapy in otherwise healthy individuals was reported in several studies [152,177,178]. An increased risk of osteonecrosis conferred by testosterone therapy was also reported, especially in relation to the heterozygosity for FV Leiden [152], but many patients had more than one prothrombotic trait. It is worth mentioning that patients with coagulation and/or fibrinolysis abnormalities usually developed bilateral osteonecrosis during testosterone therapy, which was sometimes multifocal (involvement of three or more anatomic sites) [152,177]. 

Testosterone can promote thrombogenesis by multiple mechanisms. Exogenous testosterone increases blood viscosity, platelet activity, thromboxane A2 level and subsequently the risk of blood clot formation [179]. In normal conditions, a small amount of adrenally generated androstenedione is converted to estradiol [180]. In men given exogenous testosterone, a high serum estradiol level may result from the aromatization of testosterone and estradiol-induced thrombophilia superimposed on familial thrombophilia can lead to thrombosis [181]. Since both exogenous estrogen and testosterone can interact with previously undiagnosed hereditary coagulation/fibrinolysis abnormalities to produce thrombotic events, an ONJ of uncertain etiology which occurs during hormone therapy should be investigated for the existence of a hereditary prothrombotic substrate.

Deficiencies in proteins C and S are strongly correlated with osteonecrosis, especially of the large joints [114,115]. In patients with ONJ, these hereditary thrombophilia traits were considered to be contributors to the disease, usually in association with other coagulation or fibrinolysis defects [7,106].

The G20210A polymorphism in the prothrombin gene is not an important contributor to osteonecrosis [100,101,102,103,119], being identified in only one patient with ONJ [109].

The data are also scarce and contradictory regarding the MTHFR C677T gene polymorphism’s role in osteonecrosis. Its association with ONJ was reported in only one case, that of a patient with multiple thrombophilia/hypofibrinolysis traits [104].

Although not reported in patients with ONJ, high FVIII and vWF levels are currently recognized as contributors to osteonecrosis. An association between high FVIII levels and osteonecrosis was highlighted in several studies [162,163,164,182]. Of 49 patients with non-traumatic ONFH, 12% had high FVIII levels [162]. In another study, of 71 patients with idiopathic ONFH and 62 patients with secondary ONFH, 27% and 26% of patients, respectively, had high levels of FVIII, independent of smoking–diabetes–hypertension-mediated inflammation [163]. Moreover, in patients with idiopathic multifocal osteonecrosis, high FVIII levels were found in 24% of patients [165] up to half of them [164]. 

To date, vWF has been linked to both, arterial and venous thrombosis [92,93]. Due to its ability to interact with both FVIII and platelets, vWF can recruit platelets to sites of vascular injury [92] and protect FVIII from degradation by APC [183]. Still, its involvement in osteonecrosis is less known. The first study reporting an association between vWF and osteonecrosis included 68 patients with non-traumatic ONFH and 36 healthy controls [114]. High levels of vWF were found in 23.5% of idiopathic and 23.5% of secondary ONFH patients and in none of the controls. High levels of FVIII and vWF could be potential contributors to ONJ; therefore, this relation warrants further study. 

Impaired fibrinolysis is an important contributor to the thrombotic occlusion of intraosseous vessels. In patients with osteonecrosis of large joints, hypofibrinolysis alone and mixed thrombophilia/hypofibrinolysis were found in up to 50% and 22% of patients, respectively [5]. Hereditary hypofibrinolytic traits were frequently highlighted in ONJ patients as well, mainly high levels of Lp(a) and PAI-1. These fibrinolysis abnormalities are rarely isolated. More commonly, they are associated with each other or with hereditary/acquired thrombophilic traits. In the largest available study, 73% of patients with ONJ had abnormal coagulation–fibrinolysis parameters, with the high level of Lp(a) being the most common hypofibrinolytic abnormality identified [107].

So far, the available data support the involvement of combined coagulation and/or fibrinolysis abnormalities in the etiopathogeny of ONJ. Beyond the hereditary abnormalities of coagulation and/or fibrinolysis that lead to a prothrombotic environment, acquired systemic diseases can associate with and act as potent contributors to osteonecrosis. The presence of the antiphospholipid syndrome (APS), an acquired systemic autoimmune disease characterized by recurrent thrombosis and the persistent presence of antiphospholipid antibodies, was most commonly associated with osteonecrosis. Antiphospholipid antibodies (lupus anticoagulant, anticardiolipin antibodies and anti-β2-glycoprotein 1 antibodies) create a prothrombotic environment by binding to various phospholipids on components of the coagulation system. This results in platelet activation, the up-regulation of the tissue factor expression on the surface of endothelial cells, the suppression of the activity of the tissue factor pathway inhibitor, and a reduction in activated protein C activity and fibrinolysis [184,185]. 

APS is a strong risk factor for both venous [186,187] and arterial thromboembolic events, including ischemic stroke [188] and myocardial infarction [189]. In the largest study focusing on thrombophilia/hypofibrinolysis prevalence in patients with idiopathic osteonecrosis, high levels of anticardiolipin antibodies (ACLA) significantly correlated with the presence of bone disease [105]. Moreover, in patients with multifocal osteonecrosis, the presence of ACLA was a common finding [165]. In patients with ONJ, the association between hereditary thrombophilia/hypofibrinolysis and acquired thrombophilia was not uncommon. Up to 33% of patients with ONJ had ACLA [106]. In one case report, the lupus anticoagulant contributed to ONJ, along with the hereditary thrombophilic and hypofibrinolytic traits [105]. 

Hereditary thrombophilia and hypofibrinolysis coexist in multiple and various ways. An individual may have one or more coagulation abnormalities, one or more fibrinolysis abnormalities, or associations between the two. Although the thrombotic risk is expected to increase in patients with multiple abnormalities, the magnitude of this phenomenon is still unknown. Moreover, when an acquired hypercoagulability state or exogenous factors overlap with the hereditary thrombophilia/hypofibrinolysis state, the risk of osteonecrosis is expected to further increase; even in this case, we do not have data to indicate the multiplication factor.

The aforementioned evidence shows that ONJ usually occurs when systemic and local risk factors act synergistically to compromise the circulation in the bone marrow. Ischemic and hypoxia-related stressors negatively impact the homeostasis of bone resorption and formation, alter jawbone metabolism, favor the occurrence of osteonecrosis and accelerate its progression. Local factors systemically amplify the molecular mechanisms that are already active, behaving as “triggering” events for coagulation with subsequent thrombosis. Chronic inflammation and immunogenic reactions to xenobiotics in jawbones may amplify the underlying thrombophilic or hypofibrinolytic disorder, thus perpetuating osteonecrosis [7].

Chronic or recurring infections are associated with the activation of the complement cascade. C4a-binding proteins produced during inflammation bind to free protein S, leading to a deficiency of free protein S. Less activated protein S leads to less protein C activation given that protein S is a cofactor for protein C. When the activation of protein C is impaired, an insufficient inactivation of factors VIIIa and Va occurs, thereby creating a prothrombotic environment. Moreover, activated protein C is a key player in fibrinolysis by neutralizing PAI-1 [190]. Local excessive fibrin formation creates microthrombi that block small vessels and produce microinfarctions. This process is enhanced by heritable deficiencies in coagulation and/or fibrinolysis or by exogenous factors such as estrogen or testosterone that interfere with these two processes [190]. It was emphasized that, even when the necrotic tissue is eliminated, systemic risk factors can continue to generate thrombosis in areas with already impaired vascularity [106]. These events further impair cellular health and neoangiogenesis, which are vital to normal bone repair and regeneration processes.

The synergistic therapy—local and systemic—of ONJ in patients with thrombophilia/hypofibrinolysis finds its support in the data presented so far. Long-term anticoagulation proved to be effective in thrombophilic patients with pre-collapse primary ON of the knee [127] and hip [105,116,154,155,158]. Based on the positive experience of anticoagulant therapy in patients with ONFH and hereditary thrombophilia/hypofibrinolysis, a similar approach was attempted in patients with an ONJ and hereditary prothrombotic status. However, the results were modest. Pain relief was achieved in not more than 60% of patients [118] and drug-related somatic side effects were frequent in patients treated for hypofibrinolysis. As systemic treatment is burdened by risks and complications, this therapeutic approach does not seem useful, especially in the long term. Given that the maxilla and mandibula are more easily accessible to instrumental maneuvers than the hip or knee joints, local treatments are often chosen, especially since modern local therapies are available and have proven efficiency. Local complementary treatments with laser, ozone and autologous platelet concentrates (APCs) are implemented to improve bone healing [191]. Of the greatest interest are platelet-rich plasma, plasma-rich growth factors and platelet-rich fibrin. The results from the most comprehensive data analysis to date showed that APC use after oral surgery procedures in patients with a history of antiresorptive treatment does not influence the ONJ incidence, but improves the healing rates when used in addition to ONJ surgical treatment [192]. Regenerative medicine is now the new frontier in enhancing tissue healing, with a special focus on exosomes derived from oral mesenchymal cells [193].

The search for hereditary thrombophilia/fibrinolysis in patients with ONJ is imperative for several reasons. Firstly, thrombophilia/hypofibrinolysis states may be the underlining substrate of atypical facial pain and treatment failures encountered in patients with ONJ. A customized therapeutic approach targeting, in particular, the modifiable risk factors of ONJ will relieve the patients of persistent pain and repeated procedures. Secondly, in young patients receiving exogenous estrogen or testosterone, the diagnosis of ONJ should raise the suspicion of an underlying prothrombotic substrate. As it is well known that overlapping hormone therapy over an inherited prothrombotic substrate can lead to thrombosis, hormonal therapy will be reconsidered early and with major benefits for the patients. Thirdly, ONJ refractory to conventional medical and dental treatments represents a clinically important opportunity to search for thrombophilia/hypofibrinolysis, a pathological condition echoed throughout the body. Identifying prothrombotic status and initiating the appropriate therapy may protect the patients from subsequent systemic thrombotic events. 

Our analysis has several limitations. Firstly, the available literature is scarce. The data were collected from studies with small series of patients and case reports. Secondly, the parameters used in the evaluation of the prothrombotic state differ between studies; therefore, no general conclusions can be drawn regarding the prevalence of an individually coagulation or fibrinolysis abnormality in patients with ONJ. Despite these limitations, our analysis is valuable since, as far as we know, it stands as the most comprehensive review of hereditary thrombophilia and hypofibrinolysis traits in patients with ONJ. Thus, we provide up-to-date and systematized information that can be immediately translated into practice.

## 7. Conclusions

ONJ is a complex bone disease with a multifactorial etiology. Our analysis highlighted that ONJ refractory to conventional medical and dental treatments may have an underlining prothrombotic substrate, often represented by hereditary thrombophilia/hypofibrinolysis. Although systemic therapy to balance this prothrombotic state does not appear feasible, investigating and identifying hereditary thrombophilia/hypofibrinolysis in these patients is of the utmost importance since it triggers the prompt management of associated factors that potentiate thrombosis. Patients have clear benefits, as they are relieved of repeated dental procedures and persistent pain.
